# Screening for Patient Firearm Access Among Mental Health Care Clinicians

**DOI:** 10.1001/jamanetworkopen.2024.57295

**Published:** 2025-01-29

**Authors:** Taylor R. Rodriguez, Allison E. Bond, Shelby L. Bandel, Christopher Collins, Michael D. Anestis, Joye C. Anestis

**Affiliations:** 1 New Jersey Gun Violence Research Center, Rutgers University, Piscataway; 2 Department of Psychology, Rutgers University, Piscataway, New Jersey; 3 School of Social Work, Salem State University, Salem, Massachusetts; 4 Department of Urban-Global Public Health, School of Public Health, Rutgers University, Piscataway, New Jersey; 5 Department of Health Behavior, Society, and Policy, School of Public Health, Rutgers University, Piscataway, New Jersey

## Abstract

This cross-sectional study investigates frequency of screening for firearm access among patients by mental health care clinicians.

## Introduction

Firearm-related injuries and death constitute a major public health concern.^1^ Mental health care clinicians are well-positioned to screen patients for firearm access and have conversations about secure firearm storage (ie, unloaded, locked, and separate from ammunition).^2^ However, most research on firearm access screening focuses on medical clinicians,^3^ limiting knowledge among mental health care clinicians. This study examines mental health care clinicians’ frequency of screening, screening barriers, and confidence in implementing firearm safety in practice.

## Methods

The Rutgers University Institutional Review Board approved data collection and electronic consent was obtained for this cross-sectional study, which follows STROBE reporting guidelines. Data were collected May 2023 to February 2024 via social media and professional contacts for mental health care clinicians (eg, listservs). Eligible participants reported providing mental health care services to patients and lived in the US. Clinicians with medical degrees (eg, psychiatrists) were excluded. Of 920 initial responses, 609 responses were invalid (140 responses) or from bots (469 responses). Additional methods are available in the eMethods in Supplement 1. Internally developed outcomes included whether clinicians screened for firearm access (yes or no) and the screening method, percentage of patients screened if applicable (1-5), extent that barriers prevented screening (1-5), and confidence in implementing firearm safety in practice (1-5).

## Results

Among 311 mental health care clinicians (241 female [77.5%]; 20 Asian (20 [6.4%], 19 Black [6.1%], 21 Hispanic [6.8%], and 254 White [81.7%]; mean [SD] age, 32.3, [7.4] years among 306 clinicians with data), most worked in urban (185 clinicians [59.5%]) and hospital or organized human service (113 clinicians [36.3%]) settings (Table 1). Most individuals (227 clinicians [73.0%]) endorsed firearm access screening (ie, selected yes). Of these, most screened verbally (93.8%) and when suicide (87.7%) or violence or homicide (75.3%) risk emerged. Many screened fewer than half of their patients (104 individuals [45.8%]) and few (36 individuals [15.9%]) screened all patients (Table 2).

**Table 1.** Study Participant Characteristics

Characteristic	Participants, No. (%) [95% CI] (N = 311)
Sex	
Female	241 (77.5) [72.6-81.9]
Male	70 (22.5) [18.1-27.4]
Sexual orientation	
Heterosexual	214 (68.8) [63.5-73.8]
Bisexual	48 (15.4) [11.7-19.8]
Other minoritized identity^a^	49 (15.8) [12.0-20.1]
Race and ethnicity^b^	
Minoritized identity	57 (18.3) [14.3-22.9]
Asian	20 (6.4) [3.7-9.2]
Black	19 (6.1) [3.5-8.8]
Indigenous or Alaska Native	<5
Latine or Hispanic	21 (6.8 [4.0-9.5])
Multiracial or multiethnic	10 (3.2) [1.3-5.2]
Middle Eastern or North African	6 (1.9) [0.4-3.5]
Pacific Islander or Native Hawaiian	<5
Not listed	<5
White	254 (81.7) [77.1-85.7]
Work setting	
Hospital or organized human service	113 (36.3) [31.1-41.8]
Private practice	95 (30.5) [25.6-35.8]
Community mental health	94 (30.2) [25.3-35.5]
University training clinic	86 (27.7) [22.9-32.8]
Work location	
Urban	185 (59.5) [54.0-64.8]
Rural	46 (14.8) [11.2-19.1]
Suburban	80 (25.7) [21.1-30.8]
Firearm experience	
Current owner	53 (17.0) [13.2-21.5]
Grew up in home with firearms	108 (34.7) [29.6-40.1]
Student status	
Student	161 (51.8) [46.2-57.3]
Student degree pursued is PhD (n = 161)	107 (66.9) [59.3-73.8]
Student degree field is clinical psychology (n = 161)	128 (80.0) [73.3-85.6]
Nonstudent	150 (48.2) [42.7-53.8]
Nonstudent degree field is clinical psychology (n = 150)	62 (41.3) [33.7-49.3]
Nonstudent degree attained (n = 150)	
Doctoral level	69 (40.6) [38.2-54.0]
Master’s level	57 (38.0) [30.5-45.9]

^a^ Other included queer, gay or lesbian, pansexual, asexual, and not listed, which were grouped together due to low frequencies. Participants were able to choose 1 identity in the list.

^b^ Participant self-reported racial and ethnic identities were assessed to provide a full makeup of the sample’s characteristics. These were assessed via a single item, in which participants chose all racial or ethnic identities that applied. Participants included in the White group chose only White as their identity. Participants who chose any other identity were included in the minoritized group. Listed minoritized identities, including “not listed” for those who did not see 1 of their identities in the provided list, were all options for participants. Frequencies for each subgroup represent how many participants chose that minoritized identity, and these include overlapping groups. Participants who chose any minoritized identity were combined owing to low population numbers. As the table denotes, 57 clinicians chose at least 1 minoritized racial or ethnic identity.

**Table 2.** Descriptive Statistics of Study Variables

Statistic	Participants, No. (%) [95% CI]
**Among participants who screen (n = 227)**	
Method of screening	
Verbally	213 (93.8) [90.1-96.4]
Patient paperwork	84 (37.0) [30.9-43.4]
Record review	56 (24.7) [19.4-30.6]
Other	7 (3.1) [1.4-6.0]
Timing of screening	
Response to suicide risk	199 (87.7) [82.9-91.5]
Response to other-direct violence or homicide risk	171 (75.3) [69.4-80.6]
At intake	135 (59.5) [53.0-65.7]
Estimated proportion of patients screened, %^a^	
1-25	71 (31.3) [25.5-37.5]
26-50	33 (14.5) [10.4-19.6]
51-75	44 (19.4) [14.6-24.9]
76-99	43 (18.9) [14.3-24.4]
100	36 (15.9) [11.6-21.0]
**Among the total sample (N = 311)**	
Barriers^b^	
Lack of time	142 (45.7) [40.2-51.2]
Clients don’t need it	169 (54.3) [48.8-59.8]
Clients not interested	148 (47.6) [42.1-53.1]
Wouldn’t reduce injury/death	61 (19.6) [15.5-24.3]
It’s an intrusion of privacy	92 (29.6) [24.7-34.8]
Lack of personal expertise	156 (50.2) [44.6-55.7]
Don’t get reimbursed for it	36 (11.6) [8.4-15.5]
Clients not receptive	153 (49.2) [43.7-54.7]
Give clients ideas for harm	59 (19.0) [14.9-23.6]
Secure firearm storage importance^c^	
Very unimportant	8 (2.6) [1.2-4.8]
Somewhat unimportant	6 (1.9) [0.8-3.9]
Neither important nor unimportant	7 (2.3) [1.0-4.4]
Somewhat important	81 (26.0) [2.1-3.1]
Very important	209 (67.2) [61.8-72.2]
Firearm safety information source	
Graduate training	151 (48.6) [43.0-54.1]
Work-related training	133 (42.8) [37.4-48.3]
Media	122 (39.2) [33.9-44.7]
Personal experience	117 (37.6) [32.4-43.1]
Professional journals	84 (27.0) [22.3-32.1]
Continuing education credits	71 (22.8) [18.4-27.7]
Other	16 (5.1) [3.1-8.0]
No source	31 (10.0) [7.0-13.7]

^a^ Variable range is 1 to 5, with 1 being 1% to 25% and 5 being 100%. The overall mean (SD) was 2.74 (1.47).

^b^ Frequencies of barriers endorsed reflect participants who selected a response value of at least 2 (occasionally prevents screening). Means (SDs) reflect the total score, ranging from 1 to 5, and were 1.73 (0.99) for lack of time, 2.65 (1.31) for clients don’t need it, 1.77 (0.98) for clients not interested, 1.33 (0.77) for wouldn’t reduce injury/death, 1.50 (0.91) for it’s an intrusion of privacy, 1.98 (1.20) for lack of personal expertise, 1.23 (0.72) for don’t get reimbursed for it, 1.84 (1.04) for clients not receptive, and 1.34 (0.83) for give clients ideas for harm.

^c^ The overall mean (SD) was 4.53 (0.85).

The most endorsed screening barrier was “my clients do not need it” (mean [SD], 2.65 [1.31]). The least endorsed was lack of reimbursement (mean [SD], 1.23 [0.72]; 36 clinicians [11.6%]).

Clinicians reported that conversations regarding secure firearm storage were important (mean [SD], 4.53 [0.85]). Many individuals received firearm security information from graduate (151 clinicians [48.6%]) or work-related (133 clinicians [42.8%]) trainings. Clinicians were moderately confident in their ability to implement firearm safety practices (mean [SD], 3.70 [0.93]).

## Discussion

This cross-sectional study found that many mental health care clinicians screened for firearm access at least sometimes and were moderately confident in implementing firearm safety practices. However, most screening was targeted and in the context of risk, which may miss many people. Among firearm owners, suicide risk screening tools are unlikely to capture ideation.^4^ Indiscriminate, targeted screening inadequately identifies suicide risk, potentially overlooks individuals at highest risk, and may be influenced by clinicians’ personal beliefs. Indeed, many participants stated that their patients did not need screening. While there are many suicide screening tools, none apply to all patients, settings, or circumstances, resulting in missed intervention opportunities.^5^ Furthermore, the presence of a firearm in the home is associated with increased risk for other firearm-related (eg, accidental) deaths.^6^

Proactive screening, with every patient asked about firearm access regardless of risk, may be a promising alternative. While many clinicians reported some prior training in firearm security discussions, most endorsed lack of personal experience as a screening barrier. Establishing standardized practices and universal screening in combination with staff training may diminish the role of experience in screening decision-making.

This study is not without limitations. The study was advertised to be about firearms, introducing possible selection bias. The sample was demographically homogenous. More research is needed with diverse clinician populations and to understand how screening conversations are managed. Clinicians may have overreported screening frequency and underreported barriers. Of note, some CIs were wide, indicating less precision of some point estimates (eg, overlap in CIs for the proportion of patients screened). Future research focused on replicability and incorporating other methodology (eg, medical records) is needed.

## Notes

Supplement 1.
eMethods.

eReferences.

Supplement 2.
Data Sharing Statement
