# Supplementary material for: Screening for Patient Firearm Access Among Mental Health Care Clinicians
Source: JAMA Netw Open. 2025 Jan 29;8(1):e2457295. doi: 10.1001/jamanetworkopen.2024.57295 (PMC11780471; doi:10.1001/jamanetworkopen.2024.57295)
Supplement: Supplement 1. — eMethods. eReferences. [file jamanetwopen-e2457295-s001.pdf]

Supplemental Online Content

Rodriguez TR, Bond AE, Bandel SL, Collins C, Anestis MD, Anestis JC. Screening for patient firearm access among mental health care clinicians. *JAMA Netw Open*. 2025;8(1):e2457295. doi:10.1001/jamanetworkopen.2024.57295

eMethods.
eReferences.

This supplemental material has been provided by the authors to give readers additional information about their work.

© 2024 Rodriguez TR et al. *JAMA Network Open*.

eMethods

Measures

Internally Developed Firearm-related Questionnaire

Frequency and Method of Screening. Participants were asked: "In your current clinical role, do you ever assess for firearm access among your clients (e.g., intake paperwork, verbally, record review)?" Those who responded 'yes' were asked how (client paperwork, verbally, record review, and/or other), and when they screen (e.g., once at intake, when risk emerges). Providers who reported screening were also asked what percentage of clients they ask about firearm access (range 1-5); 1-25% (1), 26-50% (2), 51-75% (3), 76-99% (4), and 100% (5).

Barriers to Screening. The following questions were adapted from Price et al. (2007) to assess perceived barriers for asking clients about firearm access. Barriers included: lack of time, clients do not need it, clients are not interested, it would not be effective in reducing firearm injury or death, it is an intrusion on privacy, lack of personal expertise, clients would not be receptive to anticipatory guidance, not getting reimbursed for time spent, and discussing the topic would give client ideas about harming themselves/others. Responses for each ranged from 1 ("never prevents screening, 0%") to 5 ("always prevents screening, 100%)."

Confidence in Firearm Safety Practices. Assessment of confidence in implementing firearm safety practices was similar to what was used in prior literature.[1,2] Participants were asked how confident they would be in implementing the following actions in their clinical work: asking clients about the presence of firearms in the home, discussing options to increase firearm security in the home, discussing options for temporary firearm storage outside of the home, and developing a plan with clients to help them increase secure firearm storage. Participants rated

© 2024 Rodriguez TR et al. *JAMA Network Open*.

confidence from 1 (not at all confident) to 5 (extremely confident) and average responses across items were used.

Other Firearm-Related Questions. Participants were asked: “To what extent do you believe conversations about secure firearm storage are important in mental healthcare settings?” Responses ranged from 1 (very unimportant) to 5 (very important). Providers were asked what sources (e.g., graduate school) have taught them about firearm security issues (e.g., secure storage).

Demographics

The demographics questionnaire, adapted from the University of Massachusetts Boston Comprehensive Demographics Questionnaire and the American Psychological Association’s (APA) survey of Psychology Health Service Providers, has been used in prior studies and includes demographics such as race, sex, and work characteristics.[3-5] Participants were asked if they had firearms in their childhood and/or own a firearm currently.

Quality Assurance Checks

Multidimensional Personality Questionnaire-Brief Form (MPQ-BF).[6] The MPQ-BF is a 155-item questionnaire measuring personality. The validity scales, Variable Response Inconsistency (VRIN) and True Response Inconsistency (TRIN), were utilized as data quality checks in line with test developer recommendations.[6]

Chapman Infrequency Scale.[7] Thirteen true/false items infrequently endorsed by most people were included. Endorsing two or more items led to invalidation.

Bot Check Items. There were several bot checks throughout participation. These included a Qualtrics CAPTCHA item, Qualtrics duplicate response flagging, three internally developed items (e.g. put words in alphabetical order), and consistency checks.

© 2024 Rodriguez TR et al. *JAMA Network Open*.

eReferences

1. Price JH, Kinnison A, Dake JA, Thompson AJ, Price JA. Psychiatrists' Practices and Perceptions Regarding Anticipatory Guidance on Firearms. *Am J Prev Med*. 2007;33(5):370-373. doi:10.1016/j.amepre.2007.07.021

2. Traylor A, Price JH, Telljohann SK, King K, Thompson A. Clinical Psychologists' Firearm Risk Management Perceptions and Practices. *J Community Health*. 2010;35(1):60-67. doi:10.1007/s10900-009-9200-6

3. Suyemoto KL, Erisman SM, Holowka DW, et al. UMass Boston Comprehensive Demographic Questionnaire, Revised. *Behav Ther*. 2016;39:83-89.

4. American Psychological Association. *2015 Survey of Psychology Health Service Providers*. Washington, DC; 2016.

5. Rodriguez TR, Anestis JC. An Initial Examination of Mental Healthcare Providers' Big 5 Personality and Their Preferences for Clients. *Psychol Stud (Mysore)*. 2023;68(1):33-44. doi:10.1007/s12646-022-00700-8

6. Patrick CJ, Curtin JJ, Tellegen A. Development and validation of a brief form of the Multidimensional Personality Questionnaire. *Psychol Assess*. 2002;14(2):150-163. doi:10.1037/1040-3590.14.2.150

7. Chapman LJ, Chapman JP. *Infrequency Scale. Unpublished Test (Copies Available from T.R. Kwapil, Department of Psychology, University of North Caroline at Greensboro, Greensboro, NS, 27402-6170).*; 1983.

© 2024 Rodriguez TR et al. *JAMA Network Open*.
