# Supplementary material for: Screening for Patient Firearm Access Among Mental Health Care Clinicians
Source: JAMA Netw Open. 2025 Jan 29;8(1):e2457295. doi: 10.1001/jamanetworkopen.2024.57295 (PMC11780471; doi:10.1001/jamanetworkopen.2024.57295)
Supplement: Supplement 2. — Data Sharing Statement [file jamanetwopen-e2457295-s002.pdf]

Data Sharing Statement

Rodriguez. Screening for Patient Firearm Access Among Mental Health Care Clinicians. *JAMA Netw Open*. Published online January 29, 2025. doi:10.1001/jamanetworkopen.2024.57295

Data

Data available: No

Additional Information

Explanation for why data not available: Data can be made available upon reasonable request to the corresponding author.
